# Targeting the nsp2 Cysteine Protease of Chikungunya Virus Using FDA Approved Library and Selected Cysteine Protease Inhibitors

**DOI:** 10.3390/pathogens8030128

**Published:** 2019-08-15

**Authors:** Prateek Kumar, Deepak Kumar, Rajanish Giri

**Affiliations:** 1 Indian Institute of Technology Mandi, School of Basic Sciences, VPO Kamand, Himachal Pradesh 175005, India; 2 BioX Centre, Indian Institute of Technology Mandi, Himachal Pradesh 175005, India

**Keywords:** CHIKV, cysteine protease nsp2, FDA approved, molecular docking, MD simulation, PCA

## Abstract

Chikungunya virus (CHIKV) infection is one of the major public health concerns, leading thousands of cases every year in rural as well as urban regions of several countries worldwide, few to mention are India, Philippines, Indonesia, and also in American countries. The structural and non-structural proteins of CHIKV are structurally and functionally similar to other alphaviruses such as Sindbis virus, Venezuelan Equine Encephalitis virus. The precursor protein of non-structural proteins is cleaved by proteolytic activity of non-structural protein (nsp2). This multifunctional nsp2 carry out nucleoside-triphosphatase (NTPase) and RNA helicase activity at its N-terminal and protease activity at C-terminal that makes it primarily a drug target to inhibit CHIKV replication. Until the current date, no suitable treatment for chikungunya infection is available. The introduction of a new drug into the market is a lengthy process, therefore, drug repurposing is now familiar approach that cut off the time and cost of drug discovery. In this study, we have implemented this approach with Food and Drug Administration (FDA) approved drugs and known cysteine protease inhibitors against CHIKV nsp2 protease using structure-based drug discovery. Our extensive docking and molecular dynamics simulations studies leads to two best interacting compounds, Ribostamycin sulfate and E-64, with utmost stable complexes at active site of nsp2 protease. Therefore, these compounds could be suitable for inhibiting CHIKV protease activity, and ultimately the viral replication.

## 1. Introduction

The first outbreak of chikungunya virus (CHIKV) infection occurred in Tanzania in 1952 and later its occurrence was also found in most of the regions of Asia and Africa until 1960 [1]. The urban region of Thailand and India were the first to be reported for CHIKV infection during 1950–1960 and then in Indonesia and Philippines in 2011 [2]. Later, in 2013, American countries were touched too and over a million of cases were registered within a year. In India, (mostly in Maharashtra and Karnataka), 12548 and 8499 cases were confirmed in 2017 and 2018 respectively [3]. Several strains of CHIKV have been identified in different parts of the world where the African and Asian strains are mostly studied. Pain in muscle, joints, ligaments and a low number of platelets are symptoms reported during chikungunya infection [4,5]. CHIKV and Dengue viruses (DENV) share some symptoms like fever, myalgia and causes of infection such as both are caused due to bite of Aedes mosquitoes in daytime. Severe joint pain leading to polyarthralgia and arthritis in some cases make CHIKV infection different from DENV [6]. Several studies on co-infection of CHIKV and Dengue viruses (DENV) have also shown the differentiable signs and symptoms using blood samples of affected patients and resulting complications due to it [7,8]. 

**Figure 1:**
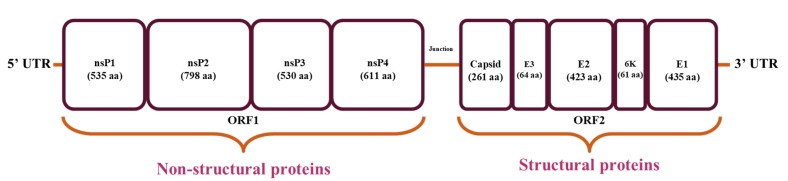
Structural representation of Chikungunya genome.

Chikungunya virus (CHIKV) belongs to the *Togaviridae* family of alphaviruses. It is transmitted by *Aedes aegypti* and *Aedes albopictus* mosquitoes and causing infection in the humans [4]. The genome size of CHIKV is ~11.9 kb with positive sense single stranded RNA and has two open reading frames (ORFs) for encoding structural and non-structural proteins [9]. Four non-structural proteins and five structural proteins are the elementary units of chikungunya virus that carry out replication. The non-structural proteins nsP1, nsP2, nsP3 and nsP4 are encoded by ORF1 (at 5′ end) and structural proteins E1, 6K, E2, E3 and C are encoded by ORF2 (at 3′ end) as shown in Figure 1, with untranslated regions at 3′ and 5′ ends of the genome [10]. 

The non-structural proteins nsP (1–3) and a free nsP4 are processed from precursor proteins which are translated by host proteins and then further cleaved into individual proteins. Although all proteins are essential for viral replication and infection to host, the nsP2 becomes more important because of its role in the separation of non-structural proteins from their precursor protein [11]. The C-terminal domain of nsP2 performs the proteolytic activity by catalyzing a reaction of deprotonation of a thiol group (-SH) at cysteine residue in the active site, which help in precursor protein cleavage, that is crucial for viral genome replication. In the course of understanding the CHIKV protease, previously, it was observed that it is a papain like protease [12,13,14], but the mutational and structure-function study by *Saisawang* et al., has shown that the catalytic dyad of CHIKV nsP2 protease is not behaving like papain during the catalytic reaction. They have even proved that the cysteine residue of the catalytic dyad in the active site is also replaceable with serine residue [15]. Overall this infers that during catalysis there are significant dynamic movements occur with other residues along with the cysteine dyad to catalyze the cleavage reaction as shown in mutational studies which is not the case in other alphavirus proteases [15]. These differential mode of catalysis by CHIKV nsP2 makes it pharmaceutically important and a challenging site for finding a suitable inhibitor against it. In our previous studies, the full length nsP2 protein was found to be less disordered with 0.5% of mean predicted percent of intrinsic disorder (PPID) in intrinsic disordered analysis of chikungunya proteome and only one short molecular recognition feature (MoRF) of ~8 residues were observed [16,17]. In other viruses like zika virus (ZIKV) and DENV, the disordered content and MoRF regions of protease domains are also comparably less than other proteins [18,19]. Viral enzymes usually have flexible cores with higher propensity of short and long disordered regions and provide dynamic functional ability to work in harsh cellular conditions [20]. The disordered regions often undergo transition from disorder to order form on binding with its partner, and therefore, are considered as important drug targets [21,22]. Based on this aspect of structural interpretation, CHIKV nsp2 seems to be a relatively rigid target for inhibitor discovery [23]. 

Several type of proteases, like serine, cysteine, aspartate, or metalloproteases have been used as key therapeutic targets for inhibitor discovery in many other viruses like hepatitis C virus (HCV), Dengue, Zika and HIV. Previously, our group has also successfully established a study on an anti-malarial drug “Hydroxyquinone” tested in vitro against Zika virus protease [24]. In past studies, cysteine protease enzymes like calpains and some cathepsins, that have their multiple isoforms in human body have also been studied as therapeutic targets for neurodegenerative diseases, and cancer therapy [25]. Calpain contributes to Alzheimer’s disease by regulating phosphorylation of the cAMP response element-binding protein (CREB) protein [26]. They form an active site with two or three catalytic residues or with metal ions. Similarly, as revealed in crystal structure (PDB ID: 3TRK), CHIKV nsp2 cysteine protease also has a catalytic dyad Cys1013 and His1083 present near the first helix at N-terminal and at a turn around β1 and β2 strands, respectively. A flexible loop close to the active site containing asparagine residue has been found that blocks the access to the substrate and substitution of this residue to alanine causes significant reduction in protease activity [11]. Currently, no adequate treatment is available for chikungunya infection. Due to its severity, it leads to other complications like arthritis and neurological disorders and causes deaths in certain cases throughout the world every year [27]. Nowadays, computational approaches have emerged on a large scale to help researchers in finding novel drugs and vaccine against a particular target in less time and cost. In addition, the “drug repurposing” or “drug repositioning” approach is quite useful to find out active molecules against different targets where most of the safety parameters were already reported for these molecules [28]. Here, in our study we have also virtually screened drugs approved by Food and Drug Administration (FDA), USA against nsp2 protease of chikungunya virus. Along with FDA approved drugs, we have also examined the efficacy of 14 cysteine protease inhibitors, acquired from SelleckChem online repository. We have further analyzed the top hits through extensive molecular dynamics (MD) studies. 

## 2. Result and Discussion

*Selection of active site:* Based on the prediction by SiteMap, we obtained five different active sites in CHIKV cysteine protease nsp2 with their respective sitescore, volume and druggability scores (Table 1). All these sites were analyzed critically, however, site 1 was ranked best due to its highest sitescore (1.007). On the other side, the predicted site 2 had the highest Dscore (1.016), donor to acceptor ratio (1.048), phobic (0.561) and philic (0.95) score for hydrophobicity and hydrophilicity of residues (Table 1). Additionally, this site contains a residue of catalytic dyad such as Cys1013 and His1083 separated by small distance, and are vastly conserved among other alphaviruses like Venezuelan Equine Encephalitis virus (VEEV). Two substrate binding residues Asn1082 and Trp1084 are also available in the active site [29]. According to Sajid et al., the catalytic dyad in cysteine proteases is surrounded by other conserved residues which help in proton transfer between the dyad. Generally, for hydrolysis, an oxyanion hole is required to form an electrophilic center, that is provided by a conserved glutamine residue in the active site of cysteine proteases [30]. In elucidation of crystal structure of VEEV nsp2 protease, *Russo* et al., has also described the importance of other conserved residues like tryptophan and asparagine residues in proteases [31]. 

**Figure 2:**
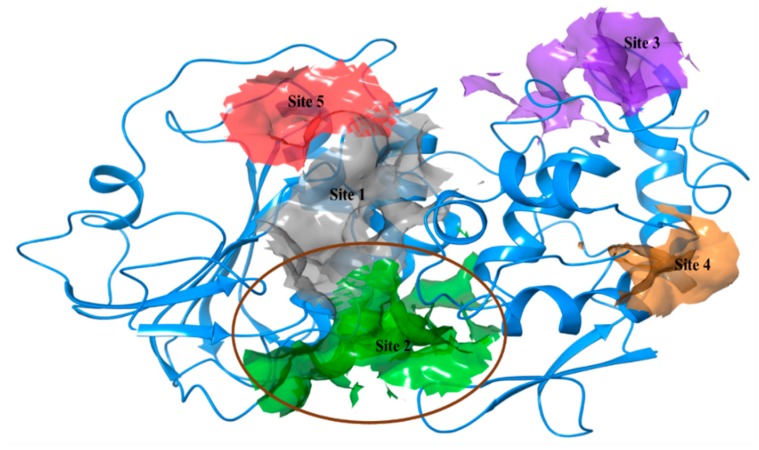
Representation of five active sites on nsp2 protease (3TRK) with respective numbers and colors predicted by SiteMap.

The predicted active site 2 has conserved residues as well as it forms a groove on the surface which is generally a property of cysteine proteases. However, predicted sites 3–5 had low scores of druggability and volume in comparison to other sites. Hence, considering all parameters, we selected site 2 in nsp2 protease for screening of nominated compounds (Figure 2). 

Molecular Docking via Extra Precision (XP) mode of Glide and interaction analysis: In virtual screening of FDA approved library of 2569 compounds (SelleckChem repository) and 14 cysteine protease inhibitors, the possible stereoisomers and tautomer were generated based on their arrangements of atoms and connectivity. All conformations of ligands were subjected to form interactions with the protein in the given grid conditions. Glide uses an empirical approach to evaluate the compounds (and their stereoisomers) and rank them after reducing the false positive results [32]. The resulted compounds from FDA library were computed with high docking scores (from ~−12 to −6 kcal/mol) and better binding energies. 

**Figure 3:**
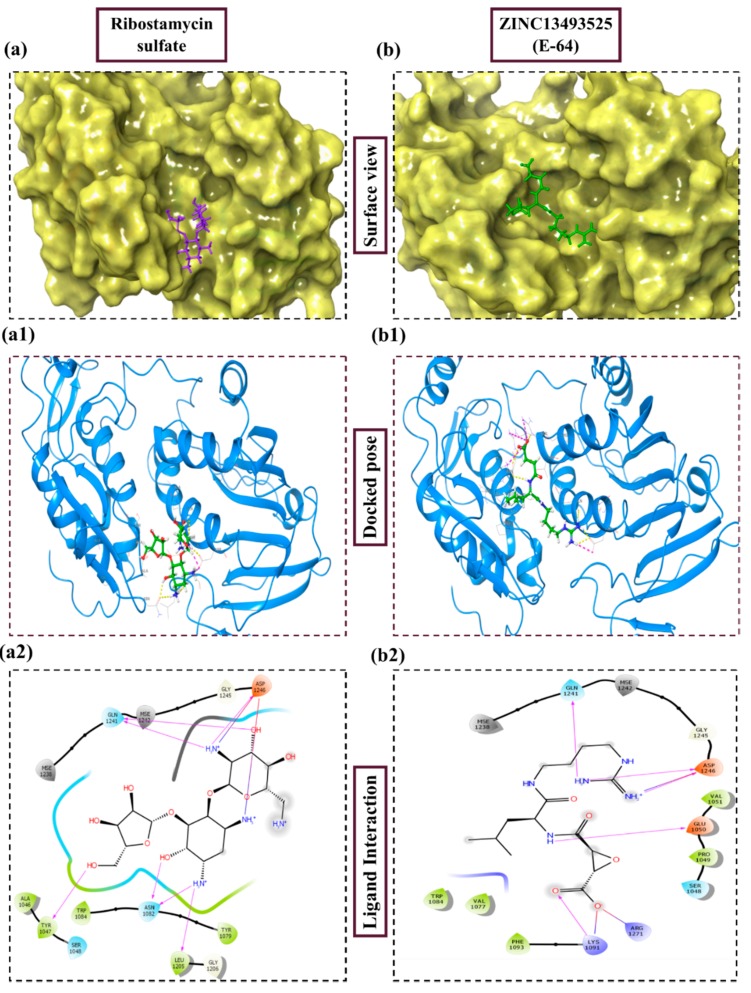
Depiction of poses within the active site of nsp2 protease: (**a**,**b**) Surface view of docked compounds binding pose of (**a1**) Ribostamycin sulfate and (**b1**) ZINC13493525 (E-64) with 3TRK. Interaction with active site residues are shown in (**a2**) Ribostamycin sulfate & (**b2**) ZINC13493525.

In Table 2 we have reported 10 compounds from FDA approved drug library with high docking scores (upto −10 kcal/mol) and MM-GBSA Δ*G_bind_* energies. The first compound, Ribostamycin Sulfate, had the highest docking score, −12.085 kcal/mol and a binding energy −30.997 kcal/mol. Ribsotamycin, an aminoglycoside derivative, is much similar to antibiotic kanamycin, that is effective against both gram positive and negative bacteria [33]. It can be either synthetically prepared or can be obtained from *Streptomyces ribosidificus.* Furthermore, Ribostamycin targets the 16S RNA and HIV-1 RNA [34]. This three-ring structured compound has interacted to CHIKV nsp2 protease via seven hydrogen bonds and two salt bridges with its ammonium ions (NH_3_^+^) and hydroxyl groups (OH) (Figure 3a2). Kanamycin, a known antibiotic obtained from Streptomyces kanamyceticus, inhibits translation in bacteria, is also resulted as one of the top best docked compounds. It binds with nsp2 protease with high docking score −10.864 kcal/mol and glide energy −64.265 kcal/mol. The binding energy calculated by Prime module, is also in significant correlation with glide docking score. These top 2 compounds from FDA library have shown binding with multiple hydrogens bonds with Asn1082, Gln1241 and Asp1246 in the active site of protein. On the other hand, Asp1246 is also involved in stabilizing the compounds with salt bridges. Similarly, other listed compounds, that are known to cure different diseases are binding in active site of nsp2 protease with hydrogen bonding, salt bridges and pi-pi stacking bonds (Table 2). Salt bridges are known as strongest bonds among all other non-covalent interactions. Formation of salt bridge catalyzes the transferring of proton from one acidic group (R-COOH) to a primary amine (R-NH_2_) in the process of binding. Majorly, the interaction of compounds via multiple salt bridges along with hydrogen bonds show that binding of previously approved FDA compounds is strong enough to get stabilized in the binding pocket and could potentially inhibit the replication and translation of viral proteins. Among all other interacting residues of nsp2 protease in a defined cutoff, Trp1084 is also forming hydrogen bonds with some compounds, which has been proved to be important for proteolytic activity in proteins [29]. In this study, the strong binding of FDA approved compounds to the key residues (Asn1082, Trp1084 Q1241 and D1246) of protease active sites have been observed. The protein stability of nsP2 protease was compared with ligand bound forms through molecular dynamics (MD) simulations. 

Docking studies of 14 cysteine protease inhibitors from SelleckChem was carried out, which have previously proven as inhibitors to other proteases like Calpain-1, Cathepsin [35,36]. Two best docked cysteine protease inhibitors are listed in the Table 2 with their structures, calculated docking scores and binding energies. The docking scores of these compounds were −8.738 and −7.08 kcal/mol, respectively. The first compound, ZINC13493525 (E-64) in the table has the highest docking score −8.738 kcal/mol and multiple interactions within the active site. It is interacted with E1050, K1091, Q1241 and D1246 via five hydrogen bonds and three salt bridges with K1091, D1246 and R1271 residues (Figure 3b2). Mainly, the hydrogen bond acceptors and donor groups are O^−^ and NH_2_^+^ of the compound. Presence of such functional groups in the compounds make them stabilize with hydrogen bonds, salt bridges, pi-pi stacking and pi-cation bonds in the pocket. The high docking score and strong interaction via multiple non-covalent bonds resemble with the binding energy, −31.659 kcal/mol and glide emodel energy −37.219 kcal/mol. E-64 has also shown binding with Falcipain-2 of *P. falciparum* via hydrogen bonding and electrostatic interactions [37]. Another cysteine protease inhibitor, Leupeptin hemisulfate, has also shown significant docking score (−7.08 kcal/mol) and binding energy (−44.461 kcal/mol) and it is interacting with Trp1084 via hydrogen bond along with Q1241 and D1246 residue. It also inhibits a wide range of enzymes like cathepsin, calpain, trypsin, etc., with significant Ki values [28,38]. Mostly, compounds are interacting with conserved residues through hydrogen bonding or other non-covalent interactions such as salt bridge formation, pi-pi stacking and pi-cation bonds with Asp1082, Trp1084, Lys1091 and Arg1271 residues (Table 2). This analysis proves that residues N1082, D1246, W1084 in the active site may have significant functions in binding of compounds along with the dyad and therefore this compound may act as potential inhibitor against the cysteine protease by binding with conserved residues in the pocket. 

Several other computational or experimental studies have given their efforts to find one-shot treatment of CHIKV infection. A review, by Subudhi et al., has summarized the compounds tested against nsp2, nsp3, capsid and other target proteins of CHIKV via in-silico or in-vitro screening. In search of inhibitors, different classes of drugs such as phenothiazines [39], flavaglines, non-steroidal anti-inflammatory drugs (NSAIDs), chloroquine [40] and epigallocatechin gallate (EGCG) [41] have been checked for blocking the entry of CHIKV. EGCG has also been reported as a potential inhibitor against ZIKV through binding with envelope protein and helicase [42,43]. Additionally, targeting the replication mechanism of CHIKV, several drugs like Ribavirin [44], mycophenolic acid (MPA) [45], 6-Azauridine [46], Suramin [47] have been tested experimentally. Apart from these drugs, monoclonal antibodies have been screened to neutralize the entry of CHIKV, membrane fusion, and inhibition of budding formation, as described by Jin et al., in their review [48]. Among all these, most have not successfully crossed in-vivo experiments or clinical trials.

### 2.1. Pharmacokinetic Properties

Although these compounds are well recognized against various protein targets, they have significant inhibition constant values with less or no toxicity, therefore, we have taken these compounds further for MD simulations. 

### 2.2. MD Simulation Analysis

The crystal structure of CHIKV nsp2 protease is 324 amino acid long, containing 32% helical and 21% beta sheets. All atom MD simulation was performed to analyze the dynamics of protein atoms and stability of the compound in the site mapped by grid for docking. Over the course of 100 ns time, both the complexes were analyzed based on their root mean square deviation (RMSD), root mean square fluctuations (RMSF), radius of gyration (Rg), and secondary structure content, that were compared with the unbound form of protein before and after the simulation. Atomic level MD simulation offers to explore the structural movement in presence and absence of ligands. The unbound form of protein and two complexes with Ribostamycin sulfate (FDA) and ZINC13493525 (E-64) (SelleckChem) were evaluated at maintained average temperature (300 K) and constant pressure for 100 ns. 

#### 2.2.1. RMSD, RMSF and Radius of Gyration (Rg) Evaluation

**Figure 4:**
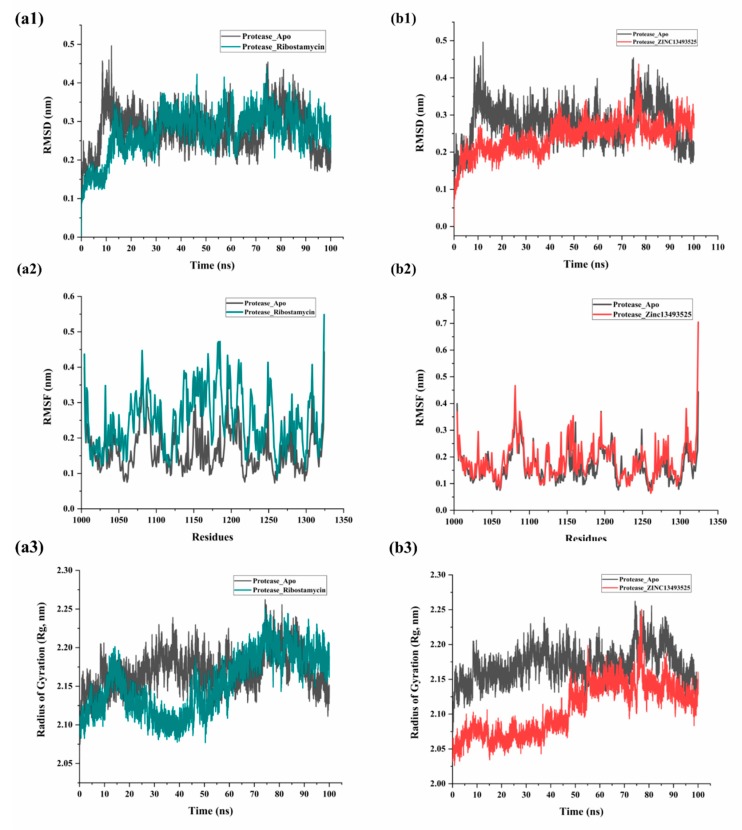
MD simulation analysis of complexes with Ribostamycin sulfate (dark cyan) and ZINC13493525 (E-64) (red) with respect to apo (black) form of CHIKV nsp2 protease: Root mean square deviation evaluation for both complexes are shown in (**a1**,**b1**) respectively. Then, RMSF in residues in simulation of 3TRK (1004-1324 residues) before and after binding with compounds are shown in (**a2**,**b2**), respectively. Similarly, compactness parameter, radius of gyration (Rg) for both complexes along with apo protein are represented in (**a3**,**b3**), respectively.

In Figure 4a, the complex with FDA compound, Ribsotamycin sulfate, showed little fluctuation with time throughout the simulation. Although the initial 10 ns trajectory was showing stable conformation for apo-protein and complex, after RMSD was increased by 1.5 Å (2 Å to 3.5 Å) it dropped again to 3 Å till 72 ns. For the Ribostamycin complex with protease, the RMSD started from 1.2 Å following a similar trend like apo-protein which further gets stabilized throughout a 100 ns period (Figure 4a1). The root mean square fluctuations (RMSF) of Ribostamycin complex were a little higher as compared to apoprotein which clearly shows that upon ligand binding the C-α atoms undergo fluctuations due to formation of hydrogen bonds and other type of noncovalent interactions. (Figure 4a2). On the other side, RMSD plots of unbound protein and ZINC13493525 (E-64) are shown in black and red colors respectively (Figure 4b1). There is a significant reduction in deviation of atoms of protein after binding to this compound with respect to apo conformation. The initial fluctuations after 10 ns were quite small as compared to apo protein and were maintained throughout the simulation period with average RMSD of 2.1 Å. In accordance with RMSD analysis, RMSF of the protein in both the conformations have shown good correlation (Figure 4b2). Similarly, we also calculated the compactness of protein structure, measured as radius of gyration (Rg). Lowering the value of Rg by increasing the compactness of the structure, means a well-folded structure. Rg of ZINC13493525 (E-64) bound protein shows lesser distance than the apo conformation. The plot (Figure 4b3) shows radius of gyration (in nm) with respect to time (in ns) which indicates that the degree of compactness was high initially (low radius of gyration) but changed due to the slight movement of ligand in the pocket of nsp2 protease. The overall Rg of the system was observed to be less than the unbound protein system. Interestingly, *Saisawang* et al. has investigated that E-64 (10 µM concentration) has little inhibition activity against CHIKV nsp2 protease via in-vitro experiments [15]. Based on our MD simulation results, the compound ZINC13493525 (E-64) is stabilizing the complex, thus, it could be used as starting backbone for further modification of functional groups to increase the inhibitory potential. 

#### 2.2.2. Principal Component Analysis and Solvent Accessible Surface Area (SASA) Prediction

**Figure 5:**
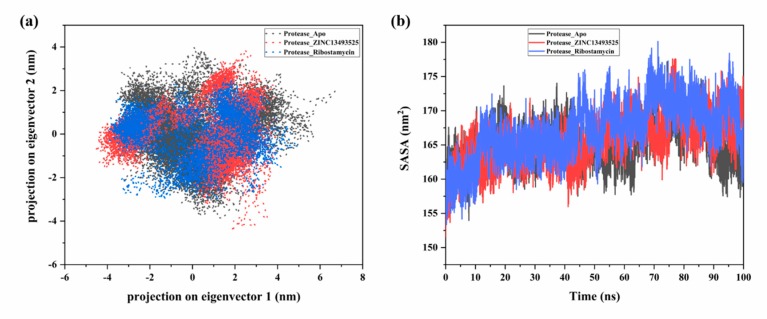
(**a**) Principal component analysis showing projections on two major components after 100 ns simulation for protease in complex with ZINC13493525 (E-64) (red) and Ribostamycin sulfate (blue) with apo protease (black). (**b**) Solvent accessible surface area of protease in bound and unbound form. Color schemes are same as (**a**).

To analyze the trajectories of all-atoms MD simulation based on their essential motions, principal component analysis or principle component analysis (PCA) was carried out to detect the conformational changes overall. It calculates the eigenvectors and eigenvalues of proteins based on a covariance matrix formed from standardized data. However, simulation trajectories generate a large number of elements to quantify the motion in the molecule. PCA reduces these numbers and generate some major factors which defines the overall motion of the protein atoms, these factors are characterized as eigenvectors in PCA [49]. In our study, we have analyzed the simulation trajectories using covar and anaeig commands in gromacs. As shown in Figure 5a, the clusters for apo and complexes are formed with two principal components (PC1 and PC2) in phase space, which accounts for major part of total elements defining principal motions of the system. In case of unbound form, the clusters were shifting from −4 to 6 nm while the movements were reduced after binding with the cysteine protease inhibitor compound (ZINC13493525). But in the other case of the eigenvector projections, they showed quite scattered clusters after binding with Ribostamycin sulfate. By this we mean also, the cysteine protease inhibitor, ZINC13493525 (E-64) may reduce the activity of protease via stable binding (Figure 5a).

In addition to this, we have also investigated the solvent accessible surface area (SASA) for all simulation systems. The protease in complex with Ribostamycin sulfate shows the larger area exposed to the solvent while the complex with ZINC13493525 (E-64) is showing comparatively lesser area (Figure 5b). ZINC13493525 (E-64) is stably bound with the protease and have less exposed surface area. These results also confirm the docking and MD simulation-based outcomes (RMSD, Rg and RMSF).

**Figure 6:**
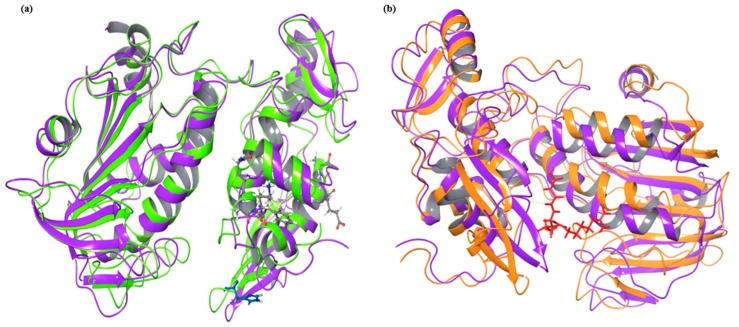
Superimposition of conformations before and after simulation in bound and unbound forms: (**a**) Apo conformations of nsp2 protease before (light green) and after simulation (violet) are overlapped with each other and residues, (**b**) superimposed conformations after simulation of unbound (violet) and E-64 bound protease (orange). The ligand E-64 is shown in red color.

We have further analyzed the structural composition before and after simulation in both, ligand bound and unbound conditions. There are significant transitions in the structure in terms of RMS distance as well as active site residue positions. In the below Figure 6a, apo conformations of nsp2 protease before simulation and after simulation are compared. The major structural changes and extending loops and turns are observed such as Ala1080 and catalytic dyad residue His1083 have gained β-strand conformation after simulation while residues in unstable helices like Glu1023, Thr1024, Ala1025, Gln1039, Ala1040, Phe1041 and Lys1042 have lost their structure after simulation. 

After MDS analysis of E-64 bound protease, we compared its frames after simulation with the frames of apo conformation after simulation. Similar with above analysis of before and after simulation, there were too structural changes after ligand binding. Due to extension of loops and structural losses, both conformations were 2.94 Å RMS distance apart after superimposition. The helix position with residue number 1039–1042 was retained after simulation in E-64 bound protease conformation but a helix in apo conformation get shorten by three residues (Leu1248, Arg1249, and Leu1250) in simulation after ligand binding. The overall RMSF was comparatively lesser than the apo conformation and other structured residues were stable with less RMSF values. 

## 3. Material and Methods

*Preparation of protein structure for screening:* The crystal structure of nsp2 protease of CHIKV is available in PDB with ID: 3RTK determined by X-ray crystallography method on resolution 2.397 Å. However, using of crystallized structure without required modification, for docking and simulation can lead to unreliable results. Therefore, the structure was first prepared in protein preparation wizard of Schrodinger by adding missing hydrogen atoms, assigning proper bond orders and removing avoidable water molecules, that were used in crystallization [50]. 

*Ligprep for Cysteine protease inhibitors and FDA approved drugs*: SelleckChem (https://www.selleckchem.com), a commercial provider of approved drugs and synthesized compounds for laboratories, provided a set of 14 compounds which has been tested against different cysteine proteases. Additionally, an updated and approved library of 2569 compounds by Food and Drug Administration (FDA) of USA was also used for screening against nsp2 protein. These compounds were prepared in LigPrep module embedded in Schrodinger suite [50].

*Active site prediction:* The crystal structure of nsP2 protease does not contain an inhibitor bound at an active site. Therefore, based on literature and prediction by the SiteMap program of Schrodinger, we identified five different active sites [51]. Site map generates site on protein structure and gives different outputs such as druggability score (Dscore), volume of site, and hydrophobic/hydrophilic ratio of residues and site score of all active sites. Based upon the Dscore, we have chosen site 2 with the highest druggability score 1.016. Using this predicted site, a grid was generated for docking of compounds with coordinates 11.84, 23.76 and 29.14 of x, y, and z axes respectively.

*Molecular Docking:* All prepared compounds were docked against nsp2 protease using the Glide module in Schrodinger [50,52]. An OPLS 2005 forcefield was used by Glide to calculate the docking scores and ranking the poses of the compounds based on their electrostatic and van der waal energies in the active site of protein. Extra precision (XP) of the Glide module was implemented for predicting best interacting poses with accurate binding energies in form of docking scores [32,53]. 

*Energy calculation using Prime:* Another scoring approach was utilized to further select the best binding poses of compounds. The calculation of binding energy and ligand strain energy was done in Prime module v3.9 of Schrodinger LLC which uses a highly accurate method MM-GBSA (molecular mechanics generalized born surface area) which runs on the OPLS2005 forcefield [54,55]. Prime has three different solvation systems; VSGB, the variable dielectric generalized Born model, water as solvent, vacuum where no solvent is used, and chloroform as a solvent. The binding energy calculation follows the following equation:

(1) Δ*G_bind_* = Δ*E_mm_* + Δ*E_slov_* + Δ*G_SA_*

where, Δ*E_mm_* is the difference in energy between bound ligand and sum of unbound ligand and protein. Δ*G_solv_* is difference in solvation energy of receptor ligand complex and the sum of solvation energy for ligand and receptor. Δ*G_SA_* is the difference in surface area energies for complex and sum of surface area energies for receptor and ligand. In this method, coulomb energy (Δ*G_coul_*), van der waals (Δ*G_vdw_*) energy, covalent (Δ*G_cov_*) and solvation binding energy (Δ*G_solv_*) were also calculated.

*MD Simulations and Principle Component Analysis (PCA)*: To evaluate the deviation between the atoms of protein in unbound and ligand bound conformation, we employed 100 nanosecond all atom MD simulation with explicit solvent water model TIP3P in Gromacs 5.1.2 [56]. The bound conformations of protein with two ligands (Ribostamycin sulfate from FDA and ZINC13493525 (E-64) from SelleckChem cysteine protease inhibitors) were investigated for their stability at nsP2 protease active site. During the method setup, V-rescale temperature coupling method, a modified Berendsen thermostat was used to stabilize the system in average temperature at 300K. This method of controlling the temperature uses velocity rescaling with a stochastic term that ensures producing all possible states of a thermally equilibrated system at a fixed temperature. In this simulation study, Charmm36 forcefield was used for generating the topology of proteins [57]. The ligand topology was generated using CGenFF server in a stream file after adding hydrogen atom in the ligands using the Avogadro program [58,59]. Energy minimization was carried out using 1000 steps run of steepest descent with conjugate gradient method. For calculating electrostatic interactions in the given periodic boundary conditions, the particle mesh Ewald method with a Fourier grid spacing of 0.16 was employed. The LINCS (LINear constraint solver) algorithm was implemented for constraining the hydrogen bonds (and angles) [60]. The constraint problem of bonds in molecules was solved by using SHAKE algorithm which alters all constraints under a specified tolerance. After accomplishing these steps, systems were then, equilibrated under NVT and NPT conditions for 100 ps of time. In the final production MD run for 100 ns timescale, all systems were subjected. For analyzing the trajectories, rms command for calculating RMSD, rmsf for calculating RMSF, gyrate command for radius of gyration and sasa for calculation of solvent accessible surface area were used. 

Subsequently, to deeply analyze the effect of ligand binding on overall conformational dynamics of protein, PCA analysis was carried out. This is a mathematical covariance-matrix based technique which generates the configurational space corresponding to vibrational modes of group of atoms in simulation trajectories [61,62]. The direction of motion was investigated by diagonal matrix of eigenvectors [63]. The GROMACS command interface was used for PCA analysis for C-α atoms using covar and anaeig commands. Chimera and VMD (visual molecular dynamics) were used for visualization and analysis of trajectories. This study was accomplished on Intel(R) Xeon(R) Gold 6130 CPU 2.10 GHz embedded in a DELL system. 

## 4. Conclusions

In this study, we have employed computational approaches to identify the binding potential of different FDA approved compounds and selected cysteine protease inhibitors against nsp2 protease of chikungunya virus. Two top hits, Ribostamycin sulfate and E-64, from both types of compound lists were examined with their docking, and molecular dynamics properties. Although the cysteine protease inhibitor, E-64 has shown little inhibitory effects against CHIKV nsP2 protease experimentally, the docking and MD analysis provides the support for stable binding at the active site which could further utilized to consider E-64 as backbone molecule for chemical modifications to improve inhibitory potential. Principal component analysis and SASA calculation also have significant co-relation with the binding results. These compounds have shown their interaction with conserved residues like Asn1082, Trp1084 and Q1241 via hydrogen bonds or salt bridge formation. One of the FDA approved compound Ribostamycin sulfate must be checked invitro for the inhibitory potential based upon our docking and simulations results. Also, these compounds could be considered as backbone molecules to further improve the binding and activity. 

## Figures and Tables

**Table 1:** Prediction of five active sites in the crystal structure of Chikungunya virus (CHIKV) nsp2 protease (PDB ID:3TRK).

Predicted Active Sites	Site Score	Dscore	Volume	Phobic	Philic	Residues
**Site 1**	1.007	0.998	215.747	0.475	1.127	I1038, Q1039, A1040, 1042, E1043, K1045, A1046, Y1047, E1055, K1191, MSE1192, N1202, L1203, E1204, I1221, T1223, P1224, R1226, V1234, D1235, MSE1238, K1239, MSE1242, L1243
**Site 2**	0.98	1.016	311.101	0.561	0.95	N1011, C1013, W1014, A1046, Y1047, S1048, E1050, V1051, N1054, L1065, Y1079, N1082, W1084, L1192, Y1201, N1202, E1204, L1205, G1206, L1207, P1208, Q1241, MSE1242, G1245, D1246, L1248, R1267, T1268, R1271, V1272, V1275, L1276
**Site 3**	0.791	0.761	134.456	0.421	1.058	G1062, Q1119, C1121, V1122, T1123, T1124, R1126, I1127, E1128, D1129, N1131, T1133, T1134, N1135, I1136, I1137, P1138, V1139
**Site 4**	0.728	0.492	38.416	0.834	1.48	N1004, F1006, V1019, L1022, E1023, I1027, K1028, L1029, W1034
**Site 5**	0.533	0.455	58.653	0.144	0.951	H1151, H1222, T1223, P1224, F1225, Q1232, Y1262, T1292, S1293, E1296

**Table 2:** Illustration of binding of Food and Drug Administration (FDA) approved compounds and cysteine protease inhibitors with CHIKV nsp2 protease.

Sr. No.	Compounds	Structure	Docking Score (kcal/mol)	MMGBSA dG Bind (kcal/mol)	Hydrogen Bond and Other Non-Covalent Interactions
***Docking with FDA drugs***					
1.	**Ribostamycin Sulfate**	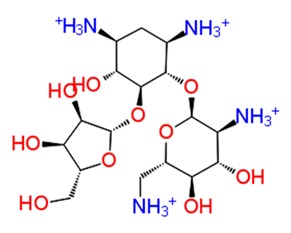	−12.085	−30.997	**H-bond**: Y1047, N1082 (2), L1205, Q1241 (2), D1246 **Salt bridge:** D1246 (2)
2.	**Kanamycin sulfate**	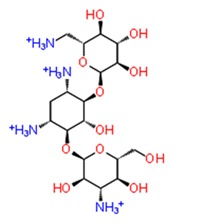	−10.864	−24.635	**H-bond:** Y1079, N1082 (2), Q1241 (2), D1246 (2) **Salt bridge:** D1246 (2)
3.	**Sennoside A**	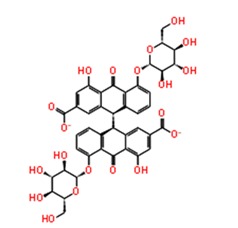	−10.404	−47.652	**H-bond:** G1090, Q1241, D1246 (2), R1271 **Salt bridge:** K1091
4.	**Amikacin sulfate**	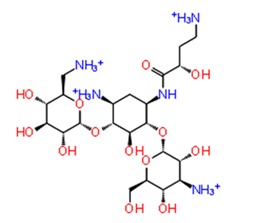	−10.389	−34.798	**H-bond:** W1084, E1204, G1245 D1246 **Salt bridge:** D1246
5.	**Dihydrostreptomycin sulfate**	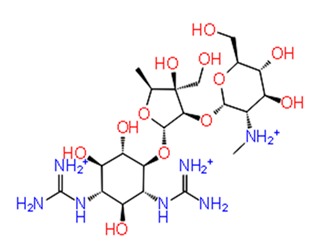	−10.36	−34.720	**H-bond:** N1082, L1205, D1246 (2) **Salt bridge:** D1246 (2)
6.	**Acarbose**	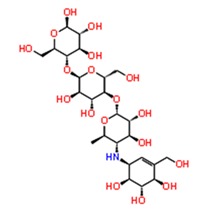	−10.2	−44.927	**H-bond:** Y1079, L1205, G1206, Q1241, D1246 (2)
7.	**Iron sucrose**	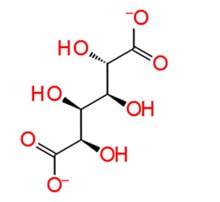	−10.13	3.473	**H-bond:** K1045, A1046 (2), L1203, K1239 **Salt bridge:** K1045, K1239
8.	**Pemetrexed disodium hydrate**	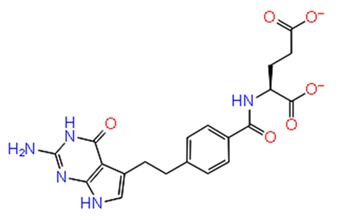	−10.077	−33.812	**H-bond:** D1246, R1267 Pi-Pi stacking: Y1079, **Salt bridge:** K1091, R1267
9.	**Ibandronate sodium**	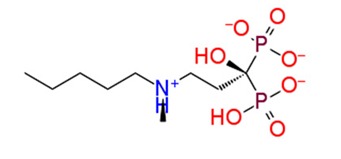	−10.045	−37.746	**H-bond:** E1050, R1267 (2), R1271 (2) **Salt bridge:** E1050, K1091 (2), R1267, R1271
10.	**Etidronate**	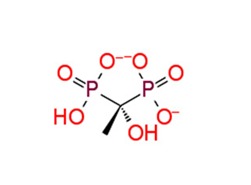	−10.014	−15.141	**H-bond:** E1050, K1091 (2), R1271 **Salt bridge:** K1091, R1267 (2), R1271 (2)
***Docking with cysteine protease inhibitors***					
11.	**E-64 (ZINC13493525)**	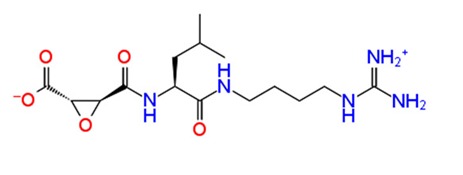	−8.738	−31.659	**H-bond:** E1050, K1091, Q1241, D1246 (2) **Salt bridge:** K1091, D1246, R1271
12.	**Leupeptin hemisulfate**	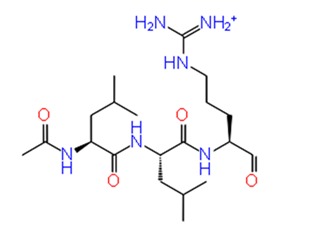	−7.08	−44.641	**H-bond:** Q1241, D1246, W1084
